# Enhancement of Adjuvant Functions of Natural Killer T Cells Using Nanovector Delivery Systems: Application in Anticancer Immune Therapy

**DOI:** 10.3389/fimmu.2017.00879

**Published:** 2017-07-27

**Authors:** Reem Ghinnagow, Luis Javier Cruz, Elodie Macho-Fernandez, Christelle Faveeuw, François Trottein

**Affiliations:** ^1^Univ. Lille, U1019 – UMR 8204 – CIIL – Centre d’Infection et d’Immunité de Lille, Lille, France; ^2^Centre National de la Recherche Scientifique, UMR 8204, Lille, France; ^3^Institut National de la Santé et de la Recherche Médicale U1019, Lille, France; ^4^Hospitalier Universitaire de Lille, Lille, France; ^5^Institut Pasteur de Lille, Lille, France; ^6^Translational Nanobiomaterials and Imaging, Department of Radiology, Leiden University Medical Center, Leiden, Netherlands

**Keywords:** natural killer T cells, adjuvant, α-galactosylceramide, CTL response, nanovaccines, dendritic cells, cancer

## Abstract

Type I natural killer T (NKT) cells have gained considerable interest in anticancer immune therapy over the last decade. This “innate-like” T lymphocyte subset has the unique ability to recognize foreign and self-derived glycolipid antigens in association with the CD1d molecule expressed by antigen-presenting cells. An important property of these cells is to bridge innate and acquired immune responses. The adjuvant function of NKT cells might be exploited in the clinics. In this review, we discuss the approaches currently being used to target NKT cells for cancer therapy. In particular, we highlight ongoing strategies utilizing NKT cell-based nanovaccines to optimize immune therapy.

## Introduction

Invariant or type I natural killer T cells (referred as NKT cells) represent a highly conserved subset of non-conventional T lymphocytes endowed with a remarkably broad range of immune effector and regulatory functions. These cells recognize foreign and self-derived glycolipid antigens presented by the monomorphic MHC/HLA class I-like molecule CD1d expressed by antigen-presenting cells, including dendritic cells (DCs) [for reviews, Ref. (1–5)]. NKT cells express on their surface a semi-invariant T cell receptor (TCR) composed by a unique TCR-α chain paired with a restricted number of β-chains. Rapidly after natural activation (inflammation, infection), NKT cells produce huge amounts of cytokines including T helper (Th)1-like (INF-γ), Th2-like (IL-4), Th17-like (IL-17, IL-22), and regulatory (IL-10) cytokines. This flexibility depends on the mode of stimulation, on the location and on the NKT cell subset challenged. Of note, NKT cells can be activated by direct TCR triggering and also *via* cytokines, without TCR engagement (5). With their ability to swiftly release cytokines, NKT cells have also the potential to lyse cellular targets following TCR recognition of lipid antigens (6). This property is important in immune surveillance against tumor cells and could be exploited for immune-based therapy. The role of NKT cells in various pathologies including cancer, infection, acute, and chronic inflammation and autoimmune diseases has been evidenced in experimental models and in humans (5). Along with their natural (beneficial or detrimental) role in pathological settings, NKT cells can also be manipulated by means of specific CD1d-restricted ligands. For instance, exposure of antigen-presenting cells to α-galactosylceramide (α-GalCer) triggers potent innate and acquired immune responses. Of particular interest is the exquisite capacity of NKT cells to promote DC maturation and, as a consequence, to trigger potent T and B cell responses (7). This unique property, and given that the CD1d/NKT axis is conserved in humans (with no HLA restriction), could be used in clinical situations, including cancer. There is a strong interest to exploit the adjuvant effects of α-GalCer or related glycolipid derivatives to develop more efficient NKT cell-based vaccines (8–10). We herein review the effects of α-GalCer in preclinical and clinical studies and discuss ongoing and future strategies that aim to optimize NKT cell-based antitumor therapy with a particular focus on nanovector delivery systems. These systems, particularly those allowing encapsulation of tumor antigens and α-GalCer derivatives (adjuvant), might realize maximal therapeutic benefit with minimal toxicity.

## Free α-GalCer in Antitumor Therapy: from Preclinical Studies to Clinical Development

Alpha-GalCer is a marine sponge-derived glycosphingolipid originally discovered in a screen for antitumor compounds (11, 12). This seminal discovery has led to the development of synthetic α-GalCer derivatives as a family of powerful glycolipid agonists for NKT cells in order to promote protective immune responses against infections and cancers (13–15). α-GalCer triggers a mixed response by NKT cells including the production of IFN-γ, a cytokine important in tumor immune surveillance and inhibition of angiogenesis. Different agonists with Th1-promoting functions (which appear to be more adapted for anticancer therapies) have been described (13, 16). Preclinical studies have highlighted the potent antitumor effect of α-GalCer and α-GalCer derivatives against solid tumors (sarcoma, melanoma and colon, prostate, and lung carcinoma) and hematological malignancies (lymphoma) (12, 17–21). Mechanisms involved include early production of IFN-γ by NKT cells and NK cells and secretion of IL-12 by DCs (20). This success has led to clinical trials in patients with advanced lung cancer. Free soluble α-GalCer was used. Unfortunately, no or low clinical benefits were reported among patients (22–24). These disappointing results might be due to the lower number of NKT cells in patients relative to healthy individuals and/or to their diminished (but reversible) activation threshold capacity (22–32). Hence, one concern in NKT cell-based therapy is the diminished NKT cell count and/or function, although this cannot be generalized to all advanced cancer patients. Various means of circumventing this potential drawback are being developed including infusion of autologous *ex vivo*-expanded NKT cells. This approach can lead to clinically relevant antitumor responses (33–39). *In vivo* transfer of NKT cells expressing chimeric antigen receptor in order to redirect their cytotoxicity against tumor cells has also been explored in preclinical studies. This approach may provide potent antitumor activity (40, 41). Moreover, the reprogramming of NKT cells to induced pluripotent stem cells and their subsequent re-differentiation into more functional NKT cells (compared with the parental cells) is opening up new avenues in this field (42, 43). Another reason that might explain disappointing clinical data relates to the uncontrolled delivery of α-GalCer, which might lead to suboptimal primary and secondary activation of NKT cells. This later issue prompted researchers to inoculate α-GalCer in a vectorized (cellular or acellular systems) form in order to better control the delivery of the active principle and to generate more efficient innate and acquired immune-based antitumor responses.

## Vectorization of α-GalCer in Cellular Systems

Cellular systems in which α-GalCer is incorporated can act as potent (NKT cell-based) cellular adjuvants. As described below, these cellular systems include DCs, non-antigen presenting cells, and cancer cells. Studies in mice have demonstrated that α-GalCer loaded in DCs has a higher ability to activate NKT cells and to trigger antitumor responses relative to α-GalCer injected in a free (non-vectorized) form (18, 44). In the same line, adoptive transfer of α-GalCer-loaded autologous peripheral blood mononuclear cells or DCs induced clinical benefits in some patients (lung cancer and head and neck cancer), an effect that correlates with IFN-γ production (23, 33, 34, 36, 45–49). Of note, adoptive transfer of autologous NKT cells along with α-GalCer-pulsed mononuclear cells or DCs led to encouraging clinical results in term of prolonged median overall survival time (35, 36, 50). This effect was associated with a significant infiltration of NKT cells into the tumor (36) Hence, this combination therapy led to significant clinical efficacy, although technical and economic issues still persist.

Taniguchi’s group was the first to exploit artificial adjuvant vectors (aAVCs) to enhance NKT cell-based antitumor responses (8). This system can induce both innate and long-term memory CD8^+^ T cell responses against cancer. For instance, inoculation (single dose) of allogeneic fibroblasts (used as a vector cell) into which tumor antigen mRNA and CD1d with α-GalCer were introduced led to a long-lasting antitumor response (51–54). The same group has designed a human aAVC consisting of embryonic kidney cells transfected with the human melanoma MART-1 antigen and CD1d and pulsed with α-GalCer. This cellular system promoted antitumor response in humanized mice (55). Mechanistically, it is likely that allogeneic cells are selectively taken up by DCs and that the subsequent cross-presentation of tumor antigens to CD8^+^ T cells and α-GalCer to NKT cell is critical in the promotion of strong and long-lasting tumor-specific cytotoxic CD8^+^ T lymphocytes (CTL) responses.

Tumor cells are rich sources of tumor antigens. However, due to the low immunogenicity of tumor antigens, combined adjuvants are requisite in order to develop cancer vaccines. Shimizu and collaborators were the first to evaluate the capacity of α-GalCer-pulsed tumor cells (melanoma) to act as a cellular adjuvant (56). Numerous studies have validated the efficacy of this strategy in therapeutical settings in the mouse system (solid tumor and hematological malignancies) (57–66). Mechanistically, inoculated α-GalCer-pulsed tumor cells are selectively taken up by DCs (as for aAVC), which have a unique capacity to cross-present antigens from dying cells. It is also possible that the killing of CD1d-expressing tumor cells by activated NKT cells leads to the release of tumor antigens and to their subsequent cross-presentation by DCs. Whatever the mechanism, it is likely that the presentation of both α-GalCer and tumor antigens by the same DC is critical in the development of the protective tumor-specific CTL-based antitumor response. Whether this strategy could be exploited in the human setting to harness cancer progression and recurrence, without inducing autoimmunity, is still unknown. Cooperative action of toll-like receptor (TLR) ligands and iNKT cells on DC function is a well-recognized phenomenon (67). Of interest, relative to inoculation of α-GalCer-loaded tumor cells alone, coadministration of α-GalCer-loaded tumor cells and TLR9 agonists augments the antitumor response (66).

Introduction of α-GalCer and tumor antigens in antigen-presenting cells has also been attempted in preclinical models. DCs expressing the mammary tumor-associated antigen Her-2 and pulsed with α-GalCer trigger potent antitumor responses (68). The use of different models of tumors revealed that this strategy was effective both in prophylactic and therapeutic settings (69). Of interest, vaccination with DCs transduced with OVA (used here as a model tumor antigen) plus CCL21, a chemokine that attracts both T cells and NKT cells, protects against OVA-expressing tumors (70). Finally, human embryonic stem cell-derived DCs genetically engineered to express CD1d can prime CD8^+^ T cells against tumor antigens (71). The potential benefit of this latter strategy in cancer immunotherapy is being studied. In conclusion, cell-based vaccines to optimize α-GalCer activity *in vivo* are promising although technical, logistical, and financial difficulties might limit the development of such vaccines.

## Vectorization of α-GalCer in Acellular Systems

### Definition of Nanovectors

Development of nanovectors (<1 μm) holds great potential for cancer immunotherapy, including antitumor vaccines (72–74). The interest of using nanosized carriers able to incorporate α-GalCer (with or without tumor antigen) to optimize NKT cell-based anticancer therapy has recently emerged. Encapsulation of α-GalCer into nanovectors might offer several advantages relative to soluble α-GalCer. This includes preferential internalization by antigen-presenting cells (due to the size), slower and sustained release of α-GalCer in CD1d-containing endosomes, and minimal side effects (due to the lower amount required for a similar biological effect). Moreover, compared to cell-based vectorization, nanovectors are less invasive and costly (no adoptive transfer). Nanovectors offer the unique opportunity to deliver both adjuvant (including α-GalCer) and tumor antigens to the same antigen-presenting cells, especially DCs (75–77). Nanovectors represent an interesting class of delivery vehicles able to induce potent and long-lasting immune responses (78, 79). Surprisingly enough, few studies have exploited this unique property to enhance the antitumor functions of NKT cells.

Nanovectors include a multiple range of particulate systems including (among others) virus-like particles, dendrimers, silica microspheres, micelles, nanogels, nanoemulsions, liposomes, carbon nanotubes, metallic nanoparticles, and polymeric nanoparticles, which include nanospheres and nanocapsules (Table 1 and not shown). The physical properties as well as the advantages and drawbacks of nanovectors are presented in Table 1. For vaccine development, a major goal is to target DCs. Uptake of nanovectors by DCs depends on several physicochemical properties including the size, shape, surface charge, hydrophobicity, and hydrophilicity of nanovectors. To target more selectively DCs, it is possible to arm nanovectors with ligands or antibodies on their surface. Among the different delivery systems for antigen encapsulation in vaccines, particularly for cancer therapy, polymeric nanoparticles have many advantages including low toxicity, high biodegradability, amenability to controlled release of the bioactive agents (antigen and adjuvant), preservation of their stability, and potential for surface functionalization (79, 80). Currently, there is a long list of polymers used to produce nanovectors including plasma albumin, chitosan, polyethyleneimine, polylactic acid, and polylactic-coglycolic acid (PLGA). PLGA is one of the most successful biocompatible and biodegradable polymers (approved for *in vivo* use by the United States Food and Drug Administration). PLGA-based nanoparticle systems are particularly interesting since they allow high antigen density, incorporation of different classes of molecules including proteins and lipids, ability to reach MHC I pathway after uptake by DCs, and slow release kinetics delivery (75, 81–85). Attempts have been made to incorporate α-GalCer in nanosized vectors, with or without tumor antigens (Table 2). Here, we detail the effect of vectorized α-GalCer in innate and acquired immune-based antitumor responses.

**Table 1:** Physical properties, advantages and drawbacks of nanovectors.

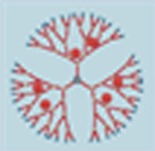	Dendrimers	1.5–14.5 nm	Chemical homogeneity, high, degree of surface functionality and versatility, controlled degradation	Multistep syntheses, elevated cost

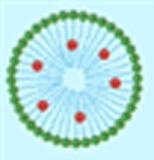	Micelles	10–100 nm	Capacity and compatibility with the loaded drug, minimized cylotoxicity	Low drug loading, low drug incorporation stability, limited targeting ability

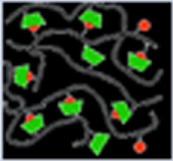	Nanogels	20–200 nm	Large Surface area, high capacity to absorb water and other biological fluids, functional modification of the surfaces to prevent rapid clearance by phagocytic cells	Difficulties to remove the solvents and surfactants (toxicity)

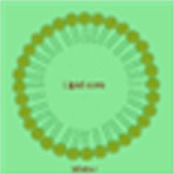	Nanoemulsions	≈100 nm	Stable structures. Large effective surface area (enhances the bioavailability of the active compound)	Special application techniques (high pressure homogenizers, ultrasonics), expensive equipment. Emulsions require large amounts of surfactants (toxic)

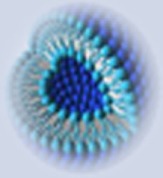	Liposomes	400 nm to 5 μm	Controlled release of the active principle (reduced side effect relative to the free form), economical production, good tolerability, specific targeting, can transport up to 10,000 active compounds Approved for clinical use	Rapid clearance due to the reticuloendothelial system low-term stability
	Multilamellar vesicles	200 nm to 1 μm		
	Large unilamellar vesicles	20 nm to 200 nm	Possibility to incorporate PEG and antibodies/ligands onto the surface to lengthen blood circulation and target immune cells	
	Small unilamellar vesicles			

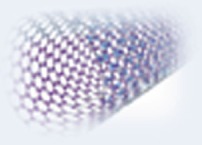	Carbon nanotubes Single-walled	Radius of up to 1 nm	Excellent chemical and thermal stability, ordered structure, high mechanical strength, high electrical and thermal conductivity, metallic or semimetallic behavior, high surface area, and bioavailability	Lack of solubility in aqueous media (may be solved by chemical modification and functionalization), potential toxic effects, aggregate formation (alteration of their general physico-chemical properties)
	Multi-walled (2–10 layers of graphene sheet)	Diameter of >10 nm		

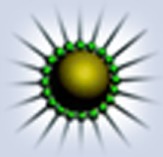	Metallic nanoparticles	5–500 nm	Biological capacity to catalyze reactions in aqueous media at standard temperature and pressure, use in molecular imaging	Toxic chemicals, high-energy requirements of production

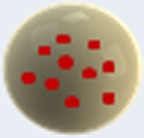	Polymeric nanoparticles Nanospheres Continuous matrix systems in which loaded drugs are generally dispersed in and entrapped by different binding systems	10 nm to 1 µm	Slower and sustained release of the active principle (adjuvant, antigens), high physical stability, simple formulation, multifunctionality, incorporation (absorption or covalent conjugation) of hydrophilic polymers (e.g., PEG/PEO-chains, polysorbate 80 polysaccharides). Cationic systems enhance DC uptake, possibility to graft ligands or antibodies to enhance the targeting	Quickly eliminated from the bloodstream (need specific design to escape the reticuloendothelial system cells)

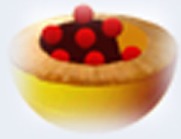	Nanocapsules Core (hydrophobic or hydrophilic) structure surrounded by a polymeric shell in which the drugs are confined	10 nm to 1 µm	Natural polymers (dextran, Chitosan, albumin, gelatin, starch) Copolymers (PFLA, PGA, PLGA) approved by the FDA for clinical use, multiple functionalization (PLGA nanoparticles) for use in cancer immunotherapy	

**Table 2:** Utilization of α-GalCer-encapsulated nanovectors to promote NKT cell activation and antitumor responses.

Nanovectors	Antigen	Targeting and NKT cell response	Antitumor response	Reference
Silica microspheres	No	Targeting of dendritic cells (DCs) and CD169-expressing macrophages (NKT) cell activation	Not tested	(86, 87)
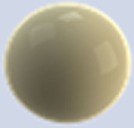				

Virus-like particles	Lymphocytic choriomeningitis virus-derived peptide gp33	NKT cell activation	gp33-specific CTL response Protection against melanoma (prophylactic setting)	(94)
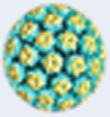				

Liposomes	No	Targeting of DCs (Mannose receptor, DC-SIGN) *via* surface oligomannose NKT cell activation (Thl biais)	Not tested	(91)
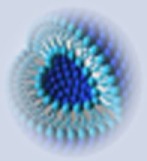	No	Targeting of macrophages (sialoadhesin CD169) *via* glycan ligands NKT cell activation (mouse and human)	Not tested	(92)
	No	Targeting of antigen-presenting cells (octaarginine-modified liposomes) strong NKT cell response	Antitumor effects (melanoma) Therapeutic setting	(90)
	Tyrosinase-related protein 2 (Trp2)	NKT cell activation	CTL response-antitumor effects Therapeutic setting	(97)

PLGA-based NPs (passive targeting)	No	Better primary activation of NKT cells (IFN-γ)	Not tested	(88, 89)
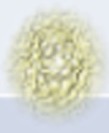	OVA	NKT cell activation	Higher CTL response relative to soluble OVA and α-GalCer and to TLR-based nanovaccine Protection against melanoma Prophylactic and therapeutic settings	(95, 96)

PLGA-based NPs (active targeting)	No	Targeting of DEC205-expressing DCs Better primary activation of NKT cells compared to soluble α-GalCer Reduced unresponsiveness of NKT cells upon restimulation	Not tested	(93)
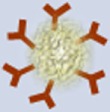	OVA	Same extent of NKT cell activation relative to NPs without OVA	Robust OVA-specific CTL response Antitumor effects (melanoma, lymphoma) Prophylactic and therapeutic settings	(93)
	Trp2 Gp100	Targeting of Clec9a-expressing DCs NKT cell activation Better primary and secondary activation of NKT cells	CTL response against tumor self antigens Antitumor effects (melanoma) Prophylactic and therapeutic settings	(107)
	Melan A	Targeting of CLEC9a-expressing DCs Expansion and activation of human NKT cells (expanded from PBMCs)	Expansion of human Melan A-specific CD8+ T cells	(107)

### Vectorization of α-GalCer without Tumor Antigen

Preclinical studies suggest that α-GalCer vectorized in nanovectors is of potential interest. This relies mainly on passive (untargeted) and active (targeted) delivery of α-GalCer to antigen-presenting cells. For instance, silica microspheres coated with lipid bilayers plus α-GalCer target mouse CD169-expressing macrophages and DCs, both cell types being critical in the primary activation of NKT cells (86, 87). Others and we have demonstrated that PLGA-based nanoparticles are internalized by DCs to promote NKT cell activation (88, 89). Another study has shown that α-GalCer incorporated in octaarginine-modified liposomes are passively taken up by antigen-presenting cells and strongly activate NKT cells. This leads to therapeutic protection against B16F10 lung metastases (90). In order to optimize the targeting of α-GalCer to antigen-presenting cells, nanovectors can be armed with ligands or antibodies that bind to specific markers. For instance, liposomes decorated with oligomannose that binds to mannose receptor and DC-SIGN target DCs *in vivo* and potently stimulate NKT cells toward a Th1 direction (91). Encapsulating α-GalCer in liposomes bearing on their surface glycans specific for the sialoadhesin CD169 strongly activates NKT cells *in vivo* (92). Our recent data demonstrate that, relative to non-vectorized α-GalCer, α-GalCer incorporated into antibody-armed PLGA nanoparticles that target DCs increases NKT cell-based innate immune responses (93).

### Vectorization of α-GalCer and Tumor Antigens

#### Passive (Untargeted) Delivery

Very few studies have been devoted so far to study the potential benefit of encapsulating α-GalCer and tumor antigens in nanosized vectors. A pioneer study from McKee and colleagues analyzed the consequences of α-GalCer and antigen co-encapsulation in antitumor responses (94). In this work, α-GalCer and the gp33 peptide derived from lymphocytic choriomeningitis virus (used as a model antigen) were incorporated into virus-like particles. This composite particle system induced a 10-fold more active gp33-specific CTL response, compared to free α-GalCer and gp33, and prophylactically protected against gp33-expressing melanoma. Mechanistically, it is likely that α-GalCer and gp33 are delivered in the endosomal compartment of antigen-presenting cells to load to CD1d and MHC Class I, respectively, thus favoring cross-presentation by DCs. Dölen and collaborators have recently demonstrated that encapsulating α-GalCer and OVA in PLGA-based nanoparticles is efficient to trigger antitumor responses (95). Of interest was the observation that the response was superior compared to TLR agonist and OVA co-encapsulation. More recently, using a similar strategy, Li and colleagues showed that the immune responses triggered by α-GalCer and OVA encapsulated in PLGA nanoparticles was longer compared to that induced by its soluble counterparts (96). Of note, both intranasal and intraperitoneal injection of nanovaccine triggered robust antigen-specific CD8^+^ T cell response. Of interest, Neumann and colleagues investigated the effect of α-GalCer and tumor antigens co-delivery on antitumor responses using a cationic liposome (97). The self-antigen tyrosinase-related protein 2 (Trp2) was used. The authors found that the liposomal formulation elicits potent antigen-specific CTL response and prevents tumor progression in a therapeutic setting. Collectively, encapsulation of α-GalCer and tumor antigens in nanovectors, including liposomes and PLGA NPs (Table 2), elicits antitumor responses in experimental models. In these settings (passive delivery), DCs and probably other antigen-presenting cells are critically important.

#### Active (Targeted) Delivery in DCs

**Figure 1:**
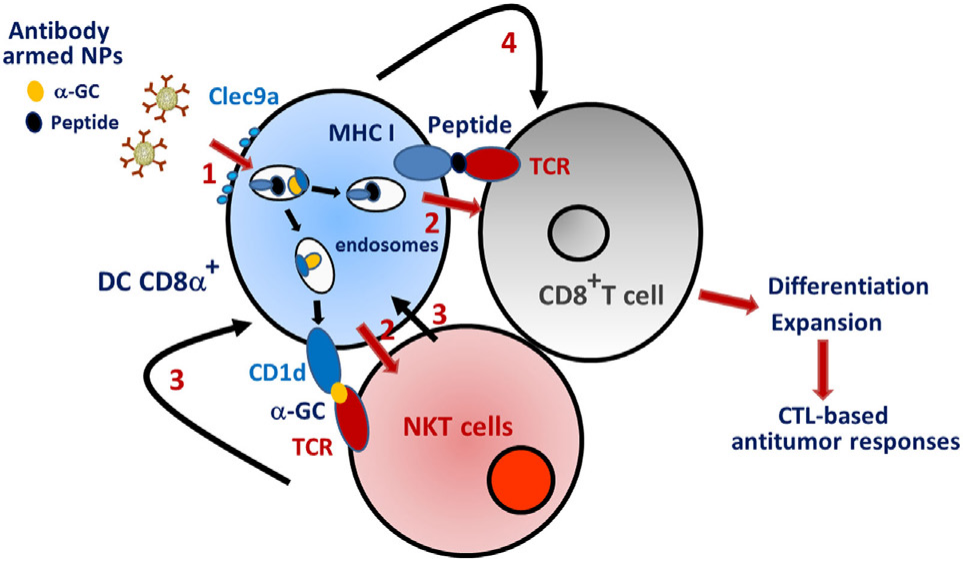
Schematic “ménage à trois” between CD8α^+^ DC, natural killer T (NKT) cells, and naive CD8^+^ T cells. (1) Anti-Clec9a-armed nanoparticles that carry α-GalCer and tumor antigen are taken up by CD8α^+^ DC *via* the endocytic receptor Clec9a. (2) The active components are delivered in the endosomes and presented *via* MHC class I (peptide) and CD1d (α-GalCer) to naïve CD8^+^ T cells and NKT cells, respectively. (3) In response to TCR triggering, NKT cells activate the maturation of CD8α^+^ dendritic cells (DCs) through cytokines and costimulatory (CD40) molecules. (4) Mature DCs transmit signals to naïve CD8^+^ T cells, which, in turn, differentiate into CTLs. (5) CTLs destroy tumor cells. Note that α-GalCer is acquired and presented by DCs that are also actively engaged in presenting peptides to T cells. This scheme does not consider reciprocal interactions between NKT cells and CD8^+^ T cells.

**Figure 2:**
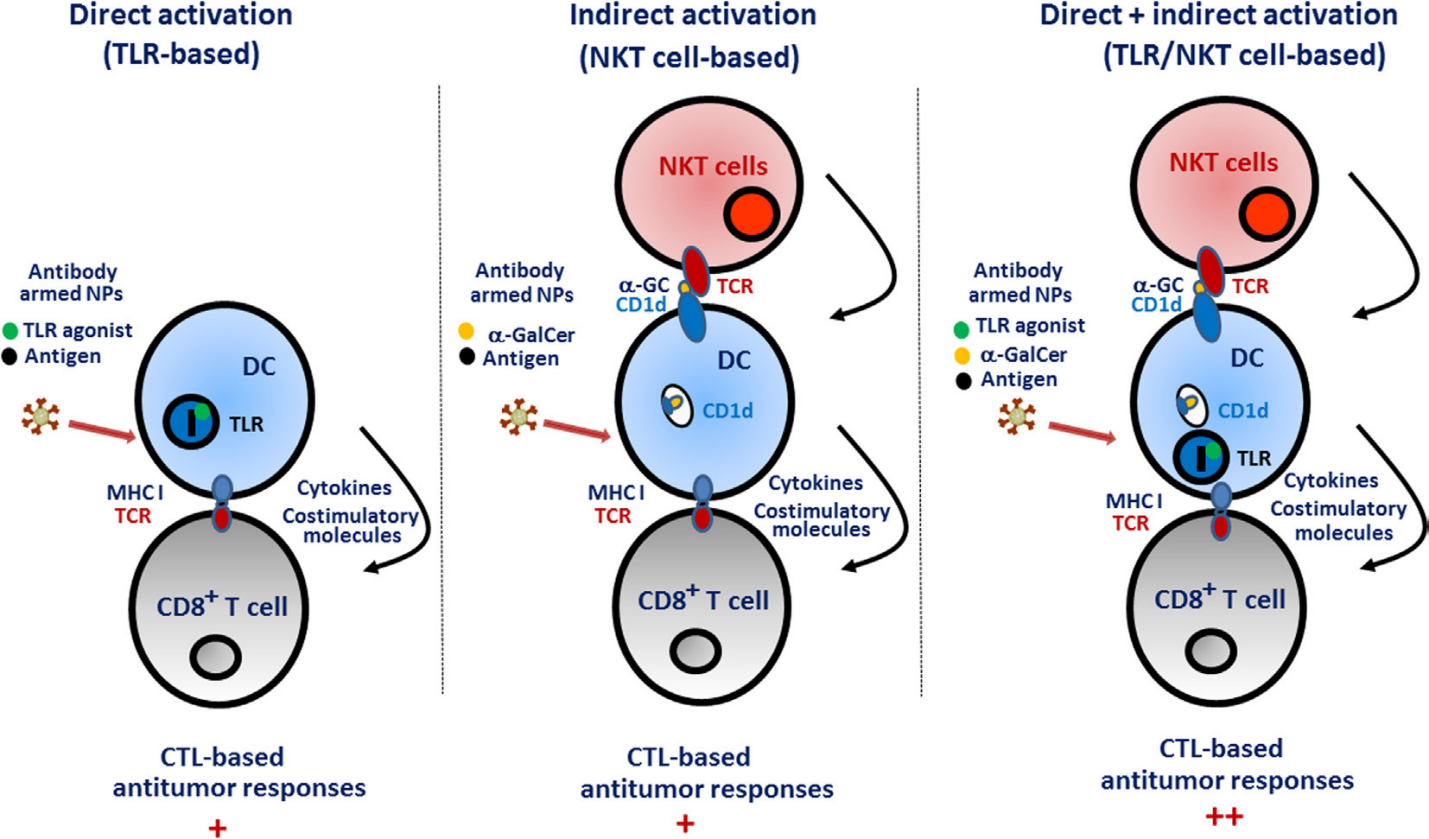
Promotion of CD8^+^ T cell responses upon direct [toll-like receptor (TLR)-based] and/or indirect [natural killer T (NKT) cell based] dendritic cell (DC) activation. Direct activation. Nanoparticles bearing TLR agonists are internalized by DCs (i.e., those that excel in cross-presentation) and activate endosomal TLRs (such as TLR3, TLR7/8, or TLR9). This rapidly leads to DC maturation and to the production of inflammatory cytokines and costimulatory molecules that in turn promote the differentiation and expansion of naïve CD8^+^ T cells. Indirect activation. In this setting, the delivery of α-GalCer in DCs leads to the exposition of the glycolipid on the cell surface in association with the CD1d molecule (at this stage, the DC is still immature). TCR triggering in NKT cells leads to the release of cytokines and to the expression of costimulatory (CD40) molecules culminating in DC maturation. In turn, mature DCs activate naïve CD8^+^ T cells. Direct and indirect activation. One may suppose that the two effects are additive, or even synergistic, to promote optimal CD8^+^ T cell responses that control tumor progression.

Our work was the first to investigate the consequences of active α-GalCer and tumor antigen delivery to DCs by means of multifunctional nanovectors. In light of the literature showing the unique ability of cross-priming DCs (CD8α^+^ DCs and BDCA3^+^ DCs in the mouse and human system, respectively) to initiate and maintain CTL responses (98–101), we decided to target this DC subset. Moreover, we and others showed that CD8α^+^ DCs are very potent to stimulate primary and secondary NKT cell activation (93, 102). Finally, the fact that NKT cells can substitute “classical” CD4^+^ Th cells to license the DCs for cross-priming represents another reason explaining our devised strategy (103). Since cross-priming DCs express specific markers on their surface, we armed PLGA-based nanoparticles with antibodies in order to target these cells *in vivo*. Although DEC205 is not entirely specific for cross-priming CD8α^+^ DCs, nanoparticles armed with anti-DEC205 antibodies and carrying both α-GalCer and OVA successfully led to antigen cross-presentation and to potent antitumor responses (93). Of interest, this strategy also led to a long lasting antigen-specific antibody response. The C-type lectin Clec9a (also known as DNGR1) is almost exclusively expressed by cross-priming mouse and human DCs and is known to confer potent CTL responses (104–106). Our recent data indicate that PLGA-based nanoparticles armed with anti-Clec9a antibodies and incorporating both α-GalCer and OVA can confer protection against OVA-expressing tumors (lymphoma) (107). We also investigated whether our vectorization/targeting strategy might break tolerance to tumor self-antigens, an important challenge for optimal antitumor therapy [for reviews see Ref. (108–112)]. Indeed, co-incorporation of α-GalCer and tumor melanoma-derived self-antigens (including Trp2) triggered a potent CD8^+^ T cell-mediated antitumor response (107). Hence, our vaccine strategy, probably by enhancing DC/NKT cell/naive CD8^+^ T cell interactions (Figure 1), abrogates self-tolerance and promotes effective antitumor CTL responses. Signals incorporated by DCs are critical to shape the functions of naive T lymphocytes, including CD8^+^ T cells. Because maturation processes of DCs due to direct innate sensor (such as TLR) signaling might be different to those triggered by NKT cells, it would be interesting to compare the efficacy of TLR-based and NKT cell-based targeted nanovaccines in cancer therapies (Figure 2). Co-administration of soluble α-GalCer and TLR agonists with antigen was shown to enhance the CD8^+^ T cell response with augmented effect on tumor progression, relative to antigen mixed with an adjuvant alone (113). Therefore, encapsulation of both TLR ligands and α-GalCer derivatives in antibody-armed nanovectors might additively or synergistically enhance the responses, a hypothesis that needs further investigations.

## Concluding Remarks and Future Perspectives

Growing evidences demonstrate that α-GalCer (or α-GalCer derivatives) might be successfully used in cancer therapy. However, innovative strategies to better manipulate the adjuvant properties and the antitumor potentials of NKT cells are required. Among them, optimization of delivery systems that contain α-GalCer and tumor antigens to optimally activate NKT cell-based immune responses remains an important goal. Cell-based vaccines that promote strong and long-lasting CTL responses offer an interesting immunotherapeutic strategy for the future although concerns still exist (cost, invasive procedure). Nanovectors that passively or actively target (cross-priming) DCs are also of clinical interest. Future studies will aim to enhance the efficacy of delivery systems in order to improve cell targeting and to optimize the delivery of the active principles (α-GalCer and tumor antigens) in the right cellular compartment. Such development will require the use of more sophisticated nanovectors to improve surgical strikes and possibly the targeting of other (DC expressed) specific molecules. Complementary approaches including strategies that boost the number/function of NKT cells in patients (transfer of functional NKT cells) and/or that aim to control immune suppression (e.g., check point blockers, immunomodulatory drugs) are of interest. Moreover, combination of NKT cell and TLR agonists might amplify the strength and the quality of the immune response in patients. An important area for future research is the development of humanized mouse models to accurately replicate the NKT cell response in humans. It is likely that, in a near future, the use of nanovector-based medicine will optimize antitumor responses for the sake of cancer patients, in combination with conventional immunotherapy.

## Author Contributions

All authors listed have made a substantial, direct, and intellectual contribution to the work and approved it for publication.

## Conflict of Interest Statement

The authors declare that the research was conducted in the absence of any commercial or financial relationships that could be construed as a potential conflict of interest.

## Abbreviations

NKT, natural killer T; DCs, dendritic cells; TCR, T cell receptor; α-GalCer, α-galactosylceramide; CAR, chimeric antigen receptor; aAVC, artificial adjuvant vector; PLGA, polylactic-coglycolic acid; CTL, cytotoxic CD8^+^ T lymphocyte; TLR, toll-like receptor; Trp2, tyrosinase-related protein 2.
